# Platelet function changes in patients undergoing endovascular aortic aneurysm repair: Review of the literature

**DOI:** 10.3389/fcvm.2022.927995

**Published:** 2022-08-12

**Authors:** Anna Burban, Aleksandra Idzik, Agata Gelo, Krzysztof J. Filipiak, Tomasz Jakimowicz, Katarzyna Jama, Marcin Grabowski, Aleksandra Gasecka, Aleksander Siniarski

**Affiliations:** ^1^Chair and Department of Cardiology, Medical University of Warsaw, Warsaw, Poland; ^2^Department of Anesthesiology and Intensive Care, Medical University of Warsaw, Warsaw, Poland; ^3^Department of Clinical Sciences, Maria Sklodowska-Curie Medical Academy, Warsaw, Poland; ^4^Department of General, Vascular and Transplant Surgery, Medical University of Warsaw, Warsaw, Poland; ^5^Department of Coronary Disease and Heart Failure, Institute of Cardiology, Jagiellonian University Medical College, Cracow, Poland; ^6^John Paul II Hospital, Cracow, Poland

**Keywords:** abdominal aortic aneurysm, endovascular AAA repair, EVAR, platelets, hemostasis, coagulation

## Abstract

Patients with abdominal aortic aneurysm (AAA) have a higher risk of cardiovascular (CV) events, which seems to be associated with disturbed platelet (PLT) function. Endovascular aneurysm repair (EVAR) is an emerging, less-invasive treatment alternative to surgical AAA repair. Both platelet function abnormalities in patients with AAA and the effect of EVAR on platelet function are poorly understood. In this review, we aim to fill the gap regarding the effect of EVAR on PLT function in AAA patients by discussing PLT function disturbances in patients with AAA, PLT function changes after EVAR, evidence from clinical studies regarding PLT function before and after EVAR, and antiplatelet or and antithrombotic treatment in patients undergoing EVAR. The goal of our review is to summarize the contemporary knowledge and initiate further studies to better understand PLT function changes in patients undergoing EVAR, optimize the pharmacotherapy before and after EVAR and further improve outcomes in this group of patients.

## Introduction

Abdominal aortic aneurysm (AAA) is defined as a maximal diameter of the abdominal aorta >3 cm, or as a focal dilation exceeding normal diameter of the adjacent arterial segment by 1.5-fold (1). AAA is said to affect 4–8% of men and 0.5–2% of women aged ≥ 60 years (2). The major concern in those patients is the risk of rupture. Evidence shows that AAA increases the risk of thromboembolic events (3). Patients with AAA have a higher risk of cardiovascular (CV) events including coronary and peripheral artery disease (PAD) (1, 3). The pathophysiological mechanisms behind increased risk of CV events remain not well-understood. AAA is associated with both higher fibrin turnover and thrombin generation, which could partly explain its prothrombotic effect (3, 4). The coagulation disorders may be due to the formation of an intramural thrombus within the aneurysm wall. Many studies have found an association between the volume of the aneurysm and the extent of coagulation disorders (3–5). Disturbed platelet (PLT) function seems to be an additional mechanism underlying the increased risk of CV events.

Endovascular aneurysm repair (EVAR) is an emerging, less-invasive treatment alternative to surgical repair, associated with lower mortality during the first 6 months, but comparable outcomes in the long-term follow-up (6, 7). The procedure can be performed with use of different types of anesthesia: general, local and regional. The meta-analysis of studies suggests that that mode of anesthesia may be associated with improved outcomes. In particular, local anesthesia appears to have a positive effect on outcome after emergency EVAR. However, no randomized trial data can prove that finding (8). However, the effect of EVAR on platelet function and coagulation disorders is unclear. Previously, we showed that the elimination of AAA from the circulation decreases PLT reactivity, which might be one of the benefits of EVAR (9). Other authors showed that EVAR promotes systemic inflammatory and prothrombotic response by triggering the cytokine release from the intramural thrombus of the aneurysm (3, 4, 10). Thus, the effect of EVAR on PLT function is to be elucidated. Patients after EVAR receive antagonists of the platelet P2Y_12_ receptors (clopidogrel). Based on the insight from CV patients, it has been suggested that low PLT reactivity during the treatment with P2Y_12_ antagonists is associated with bleeding events, whereas high on-treatment PLT reactivity is associated with thrombotic events (11, 12). However, the percentage of patients with low and high PLT reactivity after EVAR is unknown.

In this review, we aim to fill the gap regarding the effect of EVAR on PLT function in AAA patients by discussing (i) PLT function disturbances in patients with AAA, (ii) PLT function changes after EVAR, (iii) evidence from clinical studies regarding PLT function before and after EVAR, and (iv) antiplatelet treatment in patients undergoing EVAR. The goal of our review is to summarize the contemporary knowledge and initiate further studies to better understand PLT function changes in patients undergoing EVAR, optimize the pharmacotherapy before and after EVAR and improve outcomes in this group of patients.

## Platelet function disturbances in patients with abdominal aortic aneurysm

**Figure 1:**
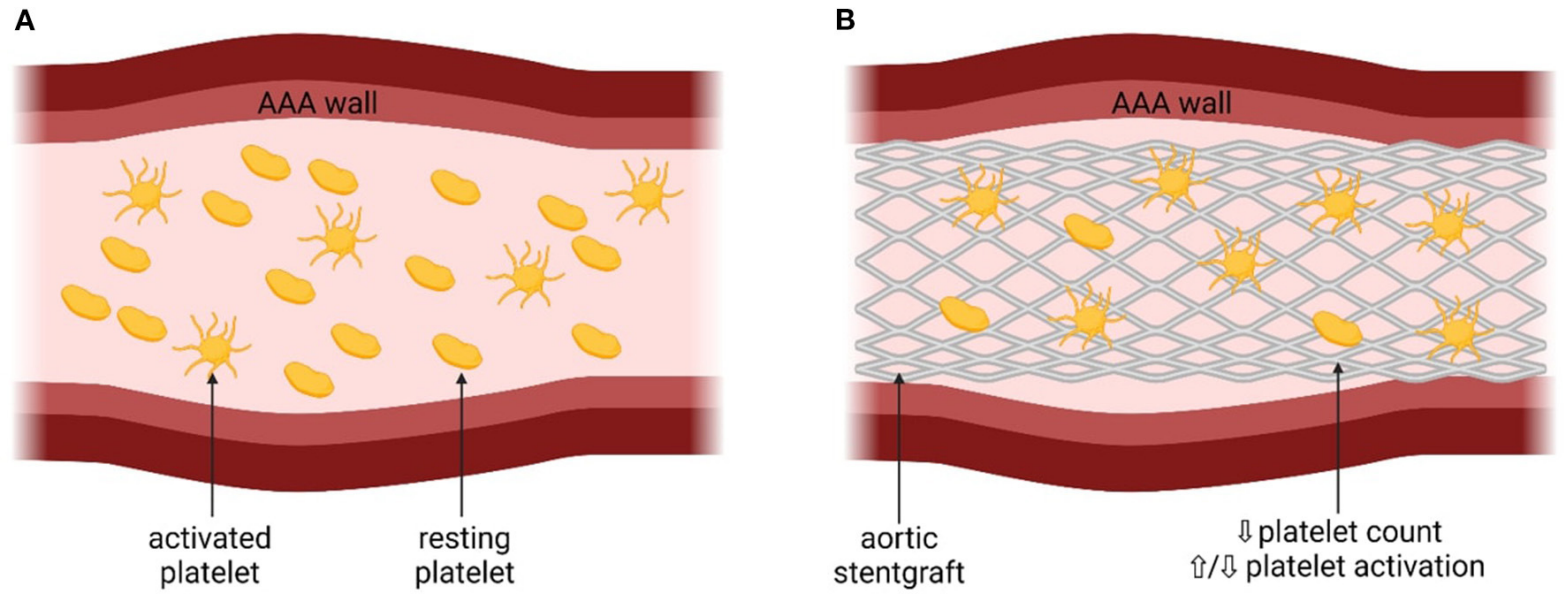
The mechanism of platelet activation associated with abdominal aortic aneurysm. AAA, abdominal aortic aneurysm; eoxPL, enzymatically oxidized phospholipids; sP, selectin – surface P-selectin; PF4, platelet factor 4; aIIbβ3, platelet integrin aIIbβ3; CD40L, CD40 ligand. **(A)** Platelets before the insertion of the stentgraft, with the presence of AAA. **(B)** Platelets after the insetion of the stentgraft.

The presence of AAA is associated with platelet function disturbances due to chronic proinflammatory response (3), including increased PLT activation and decreased PLT count (3, 13–15). The mechanism of platelet activation is shown in Figure 1.

Presence of AAA may lead to the disturbances in the hemodynamics in the aorta and therefore increase PLT activation (16). Indeed, in patients with AAA, increased PLT activation was observed, reflected by higher concentrations of soluble P-selectin and glycocalicin, compared to control patients (with symptomatic carotid artery stenoses or after AAA repair) (13–15). The prothrombotic state associated with increased PLT activation may increase the risk of CV complications in patients with AAA, although data derived from large-scale studies with the long-term follow-up are unavailable (3). It has also been speculated that the size of the aneurysm could influence PLT activation (5, 14). Significant positive correlation was found between the aneurysm size and the extent of PLT activation both in patients with and without Marfan disease, defined as dilation-dependent platelet activation (5, 14). Another prothrombotic mechanism in the development of AAA-associated CV complications has also been proposed, based on the enzymatically oxidized phospholipids (eoxPL) (17). In a murine model and in harvested human AAA samples from six male, it was showed that AAA *per se* did not cause PLT activation, but rather the procoagulant PLTs exposing eoxPL regulate AAA development through interactions with clotting factors (17). EoxPL were found in thrombus and AAA wall and delivered a procoagulant surface for binding and activation of clotting factors, promoting AAA development (17) (Figure 1). On the other hand, the presence of eoxPL somewhere else than at the AAA site diverted coagulation factors from the lesion and prevented aneurysm development (17). This highlights the complex influence of eoxPL on AAA development, showing that the modulation of the delicate balance between bleeding and thrombosis within the vessel wall or circulation could either induce or prevent the development of AAA (17).

PLT inhibitors such as acetylsalicylic acid (ASA) or P2Y_12_ receptor antagonist (clopidogrel) have an important role in reducing AAA progression, however their impact on the risk of AAA rupture is not yet well-established (18, 19). In one murine model clopidogrel has been shown to decrease the expression of the inflammatory cytokines, inhibit changes in AAA expansion and suppress the degradation of elastic lamina (18). The histopathological findings revealed a significant reduction in the macrophage infiltration and decreased release of matrix metalloproteinases (MMP). Overall, the treatment with clopidogrel caused a significant−47% reduction in AAA formation, however it did not influence the risk of rupture when compared to the control group (without P2Y_12_ inhibitor) (18). Another murine experiment studied the effect of both ASA and clopidogrel on established aneurysms (19). The results revealed a significant reduction in mortality associated with AAA rupture (ASA 0% vs. placebo 50%; clopidogrel 0% vs. placebo 47%; *P* < 0.01). It was further validated during the same experiment in human subjects in the retrospective analysis of 1,578 patients (19). It was demonstrated that both ASA and clopidogrel decreased the levels of active matrix metalloproteinase-2 (MMP-2), matrix metalloproteinase-9 (MMP-9), cytokines, plasma concentrations of PLT factor 4 and components of the plasminogen activation system in mice (19). Interestingly, a different murine study showed that the use of frequent platelet infusions had an anti-inflammatory effect on AAA development and reduced its formation by 52.38% (20). Furthermore, platelet infusion was found to decrease the inflammatory response by reducing the levels of MMP-2, MMP-9 and elastic lamina destruction, which contributed to slower aortic expansion. (20) Despite the use of ASA, some level of PLT activity associated with AAA remains unchanged (21). This fact is partially explained by a recently-discovered mechanism of regulating biochemical PLT activation associated with a membrane olfactory receptor 2L13 (OR2L13). Transcriptomic profiling of PLT from patients and mice with AAA showed that OR2L13 are involved in regulating PLT activation and AAA size progression by mediators of the aortic remodeling (21). In patients with AAA, both increased expression of OR2L13 and an upregulation of their signal transduction pathway was observed. A molecule which activated OR2L13 was identified–it limited AAA growth and platelet activation, therefore being a potential antiplatelet therapeutic agent (21).

Regarding low PLT count observed in patients with AAA, it was shown that activated PLTs which did not aggregate at the site of an aneurysm were subsequently removed from the circulation by the reticuloendothelial system (13). Consequently, about 10% of asymptomatic AAA patients had PLTs count below the normal range, and the mean platelet count was lower than in control patients with carotid artery stenoses (15). Based on observation from aggregometry- and flow-cytometry- based methods, platelet activation and formation of platelet-rich thrombi is accompanied by a decrease in platelet count, since platelet aggregates are no longer detected as single events following activation by an agonist (22, 23). Hence, decrease in PLT count in patients with AAA suggest that there is increased platelet activation and destruction, most likely within the aneurysm sac.

## Platelet function changes after EVAR

**Figure 2:**
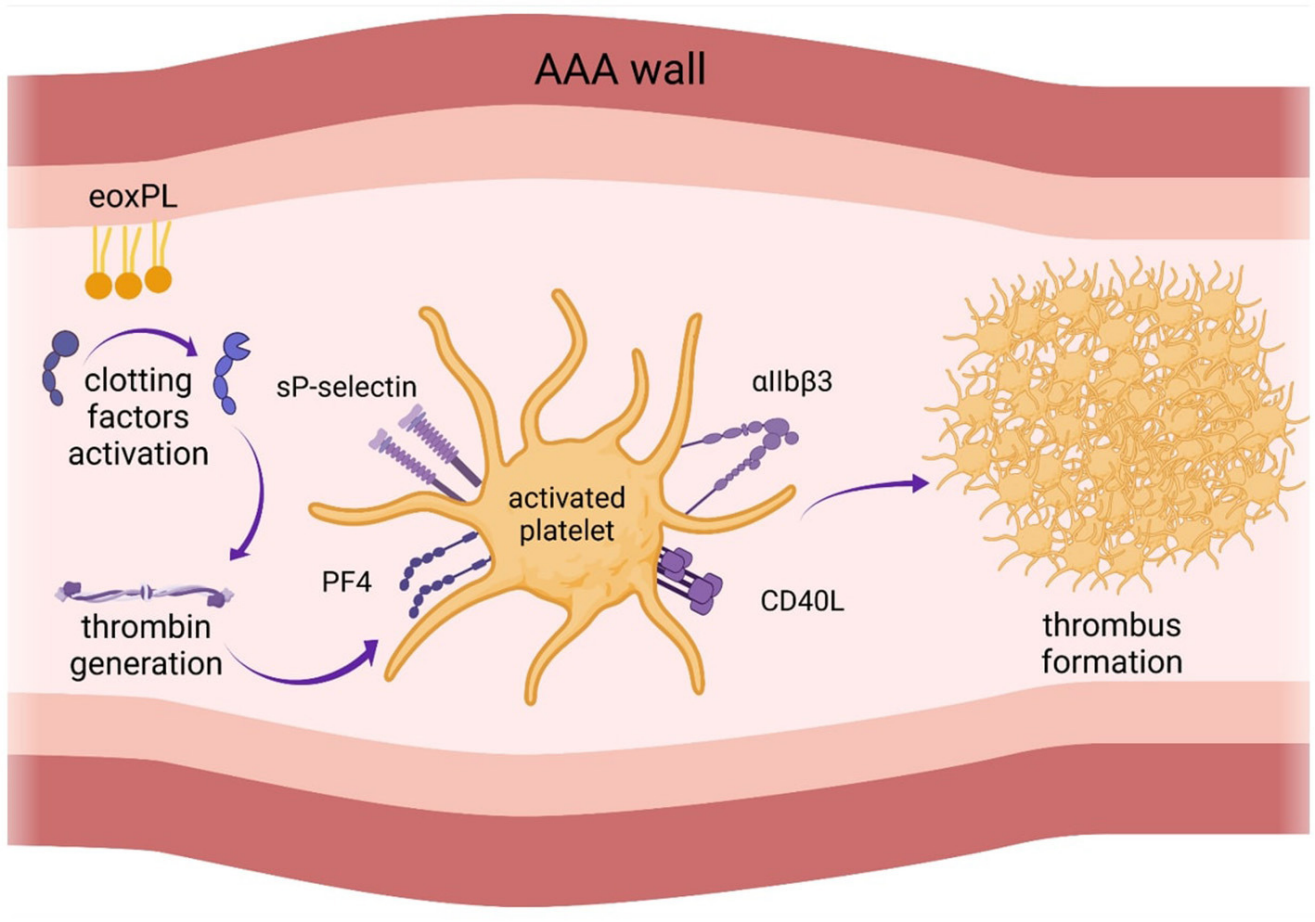
Changes in platelet function after endovascular aortic aneurysm repair (EVAR). There is consensus that platelet count decreases shortly after EVAR. However, evidence regarding the effect of EVAR on platelet activation remains contradictory. AAA, abdominal aortic aneurysm.

EVAR was shown to have a strong impact both on PLT count and activity (3, 5, 13). Generally, a postimplantation inflammatory response is common after EVAR (24). However, there are controversies in the literature regarding the magnitude and direction of these changes (3, 5, 9, 25). Generally, there is consensus that PLT count decreases shortly after EVAR. However, evidence regarding the effect of EVAR on platelet activation remains contradictory. Changes in PLT count and function after EVAR are shown in Figure 2.

Several studies have been conducted to assess changes in PLT count and platelet activation markers after EVAR, including soluble and membrane-exposed CD62p (P-selectin), CD36 (type 2 scavenger receptor for low-density lipoproteins), CD40 ligand, platelet factor 4, and glycoprotein IIb-IIIa. The results of hitherto conducted clinical studies are summarized in Table 1.

**Table 1:** Summary of change of platelet indices after endovascular aortic aneurysm repair (EVAR).

**Reference**	**Patients, number**	**Blood collections, number**	**Time point of the observed changes after EVAR**	**Change in PLT count after EVAR**	**Chance in platelet activation status after EVAR**
Abdelhamid et al. (3)	31		Up to 12 months	Not measured	Increase in P-selectin 1 month after EVAR, returned to baseline at 12 months
Liu et al. (20)	32		Up to 3 months	Not measured	Decrease in P-selectin on 3^rd^ postoperative day, increase at 3 months
Arnaoutoglou et al. (5)	50	3	Up to 3^rd^ day	Decrease	Increase in P-selectin and CD36 on 1^st^ and 3^rd^ postoperative day
Gasecka et al. (9)	50	3	1^st^, 5^th^ day	Not measured	Decrease in platelet reactivity
Gerasimidis et al. (26)	22	6	Up to 3^rd^ day	Decrease	-
Chang et al. (27)	38	3	Up to 3^rd^ day	Decrease	-
Englberger et al. (28)	15	4	1^st^ day	Decrease	-
Ikoma et al. (29)	88	5	1^st^, 3^rd^, 7^th^ day	Decrease	-
Inoue et al. (30)	205	4	1^st^, 3^rd^ day	Decrease	-
Shimazaki et al. (31)	31	5	1^st^, 3^rd^ day	Decrease	-
Yamazumi et al. (32)	36	2	3^rd^ month	Increase	-
Monaco et al. (33)	80	6	1^st^, 5^th^, 10^th^ day	Decrease	-

One of the studies assessed long-term influence of EVAR on PLT activity. The authors found that the prothrombotic diathesis normalized 12 months after EVAR procedure (3). However, it also demonstrated that the level of soluble P-selectin, a biomarker of platelet activation, was higher for 1- 6 months post-EVAR, compared to the measurement before EVAR. It decreased to the baseline level at 12 months, although the median value was still significantly higher (3). In contrast, our recent study showed that PLT reactivity decreased within 24 h after branched-EVAR compared to the measurements before the procedure (9). These discrepancies might be caused by the method of PLT function assessment and different time-points of blood collection. Most of the previous studies analyzed PLT activation indirectly by measuring the number of substances released into the bloodstream (for instance P-selectin), which are sensitive to proteolysis (34). Therefore, PLT activation based on P-selectin exposure may not reflect the original platelet activation status. In our study, a more direct method of measurement, platelet impedance-based aggregometry, was used. Aggregometry specifically measures PLT activation in response to different agonists (35) and is considered the most reliable bedside platelet function tes, with well-defined cut-off values (10). However, aggregometry also does not entirely mimic the process of platelet activation *in vivo*, since it measures platelet response to exogenous and soluble agonists (for instance thrombin receptor-activating peptide 6 instead of thrombin) (36). Hence, results from platelet function tests *in vitro* should be interpreted with caution and re-evaluated with newer generation tests. Whole blood-based perfusion systems, such as total-thrombus analyzing system which allows to monitor the process of thrombus formation under arterial shear rate (37), might be a more reliable tool to assess platelet function and may help to explain the previous discrepancies. We showed that the elimination of AAA from the circulation decreases PLT reactivity already at day 1 after EVAR and further at day 3 (9). One study demonstrated that the extent of PLT activation was associated with the type of the endograft material. Polyester grafts activated PLTs to a larger extent than polytetrafluoroethylene (5). Furthermore, inflammatory response seems stronger when using stent grafts made of synthetic polyester textile than with polytetrafluoroethylene. The length of hospital stay was longer for the patients treated with polyester stent grafts (24). Usage of contrast media also influenced PLTs activity, causing endothelial injury and increased PLT activation (13). No association between the magnitude of PLT activation and 30-day mortality after EVAR was observed (5). More studies with multiple blood collections are needed to investigate the course of PLT activation after EVAR and whether these short-term changes in PLT activation significantly influence long-term clinical outcomes including mortality.

PLT count was measured in numerous studies as an indirect index of platelet activation and consumption (5, 26–33). It has been suggested that the surgery itself is associated with platelet activation and leads to decreased PLT count, similarly as described *in vitro* (22, 23). Many studies showed a significant decrease in PLT 1-3 days following EVAR, and return to the baseline on the 7^th^-10^th^ day (5, 26–33). In conclusion, all the studies demonstrated decreased PLT count for at least 3 days after EVAR. Due to the differences in study designs and time-points of blood collection, it cannot be established when the PLT count returns to baseline or even increases following EVAR.

Currently, there are no studies showing the impact of platelet count or function following EVAR on cardiovascular outcomes including myocardial infarction and/or limb thrombosis. In our study, we showed that pre-operative platelet reactivity (measured by using ASPI, TRAP and ADP tests) was a predictor of bleeding complications (9). Nevertheless, platelet depletion (reduction >60%) following EVAR was associated with post-operative bleeding and increased mortality in one study (38). On the other hand, Inoue et al. demonstrated that the platelet count of patients with malignant type-2-endoleaks on 7^th^ day following the operation was lower than that of patients with completed EVAR or with benign type-2-endoleaks (39). The authors also showed that lower platelet count on 7^th^ day following the operation could serve as a risk factor for AAA enlargement among patients with type-2-endoleaks (39).

## Antiplatelet treatment in patients undergoing EVAR

### Treatment before EVAR

In case of smaller AAAs (<5.5 cm diameter), the conservative management along with regular imaging tests is advised (40). Currently, no medical therapy has been proven to decrease the expansion rate of AAA (41, 42).

According to the Guidelines on Management of Abdominal Aorto-Iliac Artery Aneurysms, pharmacological management in patients with AAA is in line with the treatment of patients with PAD (40). Regarding very-high CV risk, these patients should receive antiplatelet therapy, lipid lowering agents (if low-density cholesterol exceeds 2.5 mmol/L) and antihypertensives (if blood pressure is above 140 mm Hg) for the secondary prevention (40). Patients receiving statins or antihypertensive agents had 20–25% better 5-year survival rate, compared with patients not receiving such therapies (43).

ASA was showed to decrease the risk of CV events in patients with PAD, including AAA (44, 45). A retrospective study of 12 485 patients with AAA showed a 24% reduction of 5-year mortality in patients who received antiplatelet therapy (ASA, clopidogrel and dipyridamole), compared to patients without antiplatelet medications (43). However, analyzed separately, only ASA was associated with lower mortality, other antiplatelet drugs did not affect outcomes (43). Benefits of ASA in the secondary prevention of CVD have also been demonstrated in meta-analyses of clinical trials (44–46). Hence, ASA has received class I recommendation in patients with AAA. Similarly to patients with PAD, patients with AAA should receive clopidogrel as an alternative if aspirin is contradicted (44). Antiplatelet monotherapy does not increase the risk of perioperative bleeding and should not be discontinued prior to surgery (47). If patients with AAA have indication for dual antiplatelet therapy (DAPT), such as a history of PCI with stent implantation, DAPT should be administered (40, 48). If possible, EVAR should be delayed until DAPT cessation, especially in patients at high bleeding risk (40).

Currently, there is few data to support the use of newer antiplatelet drugs, such as prasugrel and ticagrelor in patients with AAA. However, the superiority of prasugrel and ticagrelor over clopidogrel in patients with coronary artery disease makes them an interesting alternative therapeutic option also in AAA patients and requires further studies.

### Treatment after EVAR

Postoperative management should focus both on the prevention of postoperative complications, such as graft thrombosis and peripheral embolization, and secondary prevention of CV events. Antiplatelet therapy can accomplish both. It has been shown that platelet activation, adhesion and aggregation plays major role in a stent-graft thrombosis (49). Hence, ASA is the basal treatment after EVAR. Six-month-long DAPT consisting of ASA and clopidogrel is recommended after fenestrated or branched EVAR (48, 50). Antiplatelet therapy also decreases the risk of PAD progression, common among patients with AAA (43). There is evidence that antiplatelet therapy significantly reduces the risk of vascular occlusion in patients undergoing peripheral angioplasty as well (50). Therefore, post-operative antiplatelet therapy is essential. Concurrently, antiplatelet therapy does not increase the risk of bleeding or endoleak into the aneurysmal sac (51). After 6 months, ASA monotherapy is the best evidence-based strategy, not only does it prevent graft thrombosis, but also reduces the aneurysm sac growth, compared to other antiplatelet or anticoagulation therapy (52). Extrapolating data derived from patients undergoing PCI to EVAR patients, the long-term low-dose ASA is as effective as the high-dose (52, 53). However, the optimal antiplatelet therapy regimens after EVAR are awaiting further investigation.

**Figure 3:**
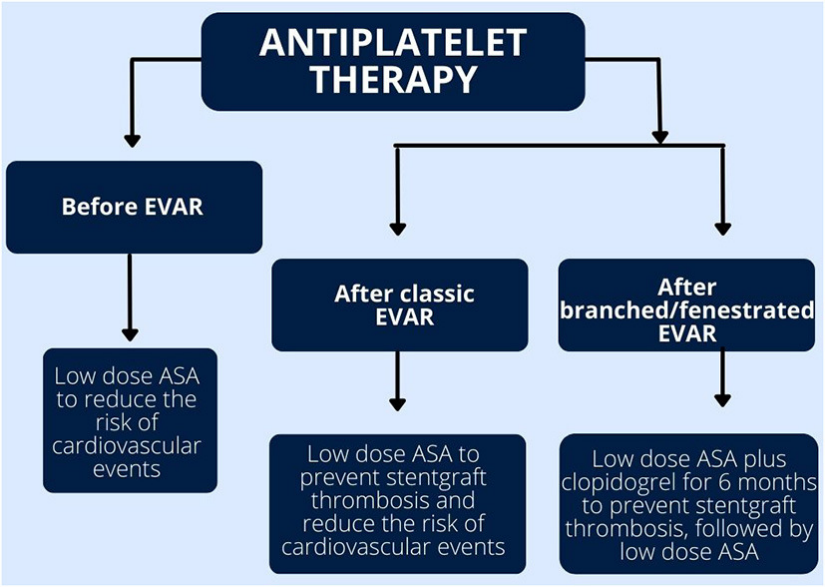
Summary of antiplatelet therapy before and after endovascular aneurysm repair (EVAR).

Currently there are no randomized controlled trials comparing the efficacy and safety of different antiplatelet drugs after EVAR. The choice of treatment is based on local experience of each clinical center (50, 54). Similarly, the best therapeutic strategy after EVAR in patients with indications for oral anticoagulation is yet to be established. Figure 3 summarized the recommended antiplatelet treatment regimens after EVAR.

## Discussion

Increased PLT activation seems to be one of the mechanisms underlying the increased risk of CV events in patients with AAA. Furthermore, it was shown to increase the risk of AAA rapture. Therefore, targeting the PLT activation could serve as a potential therapeutic goal in patients with AAA. However, despite the use of antiplatelets agents such as ASA some, residual level of PLTs activity remains unchanged, what might be associated with AAA progression (21). On the other hand, potent antiplatelets regimens such as DAPT were shown to be demonstrated with increased bleeding risk, as incidence rates for all major bleeding events were higher in dual regimens than in mono- therapy (55).

The increased PLT activation which is present in patients with AAA despite the use of antiplatelet agents can be partially explained by PLT activation associated with a membrane olfactory receptor 2L13 (OR2L13) (21). The increased expression of OR2L13 receptor in patients with AAA and the upregulation of their signal transduction pathway was observed. A molecule which activated OR2L13 was identified and was also proved to limited AAA growth and platelet activation (21). As mentioned before, AAA *per se* did not cause PLT activation, but rather the procoagulant PLTs exposing eoxPL regulated AAA development through interactions with clotting factors. Those molecular mechanisms of PLT activation are not now addressed in standard antiplatelet therapy. Therefore, those molecular mechanisms could serve in future as a therapeutic targets of action for new generation of antiplatelets regimens.

It was also demonstrated that there are many other factors which can influence the PLT activation following EVAR such as graft material or the use of contrast media. Polyester grafts activated PLTs to a larger extent than polytetrafluoroethylene (5). Furthermore, the length of hospital stay was longer for the patients treated with polyester stent grafts (24). Hence, the changes of materials which are currently used could also alter the PLT activation and influence the clinical outcomes after EVAR.

Antiplatelet therapy is the cornerstone of pharmacological treatment both before and after EVAR. However, the best treatment options, tailored to the specific mechanism and degree of platelet activation in individual patients are yet to be established. To optimize the outcome of endovascular AAA repair the treatment of underlying cardiovascular disease should be continued post-operatively what includes antihypertensive therapy, lipid modifying therapy and antiplatelets (40). The accurate PLT function changes in patients undergoing EVAR require investigation, especially with new, targeted antiplatelet therapies. This could further optimize the pharmacotherapy before and after this procedure to improve the clinical outcomes in this group of patients.

## Author contributions

AGa: conceptualization. AB, AI, and AGe: writing—original draft preparation. KF, TJ, KJ, and MG: writing—review and editing. AGa and AS: methodology and supervision. AS: formal analysis. All authors have read and agreed to the published version of the manuscript.

## Conflict of interest

The authors declare that the research was conducted in the absence of any commercial or financial relationships that could be construed as a potential conflict of interest.

## Publisher's note

All claims expressed in this article are solely those of the authors and do not necessarily represent those of their affiliated organizations, or those of the publisher, the editors and the reviewers. Any product that may be evaluated in this article, or claim that may be made by its manufacturer, is not guaranteed or endorsed by the publisher.
